# Temporal patterns of suicide and circulatory system disease-related mortality are inversely correlated in several countries

**DOI:** 10.1186/s12888-021-03159-5

**Published:** 2021-03-16

**Authors:** Marc J. Kaufman, Garrett M. Fitzmaurice

**Affiliations:** 1 Department of Psychiatry, Harvard Medical School, McLean Imaging Center, McLean Hospital, 115 Mill St, Belmont, MA 02478 USA; 2 Department of Biostatistics and Department of Psychiatry, Harvard Medical School, Laboratory for Psychiatric Biostatistics, McLean Hospital, 115 Mill St, Belmont, MA 02478 USA

**Keywords:** Suicide, Season, Temperature, Thermoregulation, Light

## Abstract

**Background:**

Nearly 800,000 suicides occur worldwide annually and suicide rates are increasing faster than population growth. Unfortunately, the pathophysiology of suicide remains poorly understood, which has hindered suicide prevention efforts. However, mechanistic clues may be found by studying effects of seasonality on suicide and other mortality causes. Suicides tend to peak in spring-summer periods and nadir in fall-winter periods while circulatory system disease-related mortality tends to exhibit the opposite temporal trends. This study aimed to determine for the first time whether monthly temporal cross-correlations exist between suicide and circulatory system disease-related mortality at the population level. If so and if common biological factors moderate risks for both mortality types, such factors may be discoverable and utilized to improve suicide prevention.

**Methods:**

We conducted time series analyses of monthly mortality data from northern (England and Wales, South Korea, United States) and southern (Australia, Brazil) hemisphere countries during the period 2009–2018 (*N* = 41.8 million all-cause mortality cases). We used a Poisson regression variant of the standard cosinor model to determine peak months of mortality. We also estimated cross-correlations between monthly mortality counts from suicide and from circulatory system diseases.

**Results:**

Suicide and circulatory disease-related mortality temporal patterns were negatively correlated in Australia (− 0.32), Brazil (− 0.57), South Korea (− 0.32), and in the United States (− 0.66), but no temporal correlation was discernable in England and Wales.

**Conclusions:**

The negative temporal cross-correlations between these mortality types we found in 4 of 5 countries studied suggest that seasonal factors broadly and inversely moderate risks for circulatory disease-related mortality and suicide, but not in all regions, indicating that the effect is not uniform. Since the seasonal factors of temperature and light exert opposite effects on suicide and circulatory disease-related mortality in several countries, we propose that physiologically-adaptive circulatory system responses to heat and light may increase risk for suicide and should be studied to determine whether they affect suicide risk. For example, heat and light increase production and release of the bioactive gas nitric oxide and reduce circulatory system disease by relaxing blood vessel tone, while elevated nitric oxide levels are associated with suicidal behavior, inverse effects that parallel the inverse temporal mortality patterns we detected.

## Background

Nearly 56 million deaths occur each year worldwide including close to 800,000 suicides [1]. While substantial progress has been made to reduce mortality associated with circulatory system diseases and cancers [2, 3], suicide deaths in the United States and worldwide continue to increase [4, 5] at rates faster than population growth [6, 7], perhaps in part because deaths from other mortality types are declining. Our slow progress at advancing suicide prevention is due in part to our incomplete understanding of the pathophysiology of suicide, which to date has resulted in the development of few treatment options [8, 9].

Important clues to better understanding suicide pathophysiology may be found by considering research on seasonal variations in mortality patterns for suicide and for circulatory system diseases, which account for 1.4 and 32%, respectively, of worldwide all-cause mortality [1]. Peaks and nadirs in suicide deaths tend to occur in spring-summer and fall-winter periods, respectively [10, 11]. Conversely, mortality peaks and nadirs for diseases of the circulatory system tend to occur in fall-winter and spring-summer periods, respectively [12, 13]. The same seasonal patterns are observed in the northern and southern hemispheres [10, 13] meaning that these temporal patterns likely are a consequence of seasonal factors such as temperature or light fluctuations. It has been proposed that physiological mechanisms that drive seasonal fluctuations in circulatory system morbidity and mortality, some of which have been identified, could be exploited to reduce circulatory system disease-related mortality [14]. By extension, physiological mechanisms activated by seasonal factors that moderate suicide risk, some of which could overlap with those driving circulatory system morbidity and mortality, may be discoverable and exploited to better identify those at increased risk for suicide.

Suicide risk increases soon after a major cardiovascular event, presumably as a consequence of increased depression, anxiety, and hopelessness accompanying cardiovascular morbidity [15, 16]. However, the effects of season were not considered in those studies and to date, a formal evaluation of the degree of temporal correlation between suicide and mortality from diseases of the circulatory system has not been conducted at the population level. Accordingly, we did this by analyzing multi-year mortality data for suicide and for diseases of the circulatory system stratified by month of occurrence. We obtained deidentified data from official death registries of northern hemisphere (England and Wales, South Korea, United States) and southern hemisphere (Australia, Brazil) countries. The dataset included more than 660,000 suicide and 12.2 million circulatory disease-related mortality cases. We conducted cross-correlation time-series analyses on data from each country to determine whether temporal correlations exist between these mortality causes. We hypothesized that temporal patterns of suicide would be negatively correlated with temporal patterns of mortality from diseases of the circulatory system.

## Methods

### Data

Multiple years of mortality data stratified by month of occurrence were obtained from official death registries in Australia [17], Brazil [18], South Korea [19], the United Kingdom [20], and the United States [21]. Data were extracted for mortality cases resulting from suicide (Intentional self-harm, ICD-10 codes X60–84), from diseases of the circulatory system (ICD-10 codes I00-I99), for all-cause mortality, and were stratified by sex. Other potentially-relevant demographics including socioeconomic, psychiatric, and medical status are not available in these databases and thus are not considered in this analysis. Data demographics are shown in Table 1.

**Table 1:** Mortality sample cases, sex ratios, and proportions of all-cause mortality

Country	All-cause N; (%♂/%♀)	Circulatory Disorders N; (%♂/%♀) %All-cause / ♂:♀ratio	Suicide N; (%♂/%♀) %All-cause / ♂:♀ratio
Australia^a^	1,360,772; (51.3/48.7)	404,082; (48.3/51.7) 29.7 / 0.93	24,590; (75.4/24.6) 1.81 / 3.06
Brazil^b^	11,129,455; (56.4/43.5)	3,103,220; (52.4/47.6) 27.9 / 1.10	98,646; (78.6/21.3) 0.89 / 3.69
England/Wales ^c^	3,008,294; (48.4/51.6)	854,477; (49.9/50.1) 28.4 / 1.00	29,255; (76.4/23.6) 0.97 / 3.24
South Korea^b^	2,455,047; (54.8/45.2)	531,094; (47.2/52.8) 21.6 / 0.89	126,633; (69.6/30.4) 5.16 / 2.29
United States^b^	23,860,169; (50.6/49.4)	7,369,083; (50.4/49.6) 30.9 / 1.02	386,536; (77.9/22.1) 1.62 / 3.52
Totals (N)	41,813,737	12,261,956	665,660

Years included: ^a^2009–17; ^b^2010–18, ^c^2010–15

### Statistical analysis

First, we considered within-country seasonality of circulatory disease-related and suicide mortality counts separately. Second, we considered within-country cross-correlation between these two mortality types over time. Parameters of seasonality within countries were estimated using a Poisson regression variant of the standard cosinor model. The cosinor regression model captures a seasonal pattern using sine and cosine terms that together describe a sinusoidal pattern [22]. In the cosinor model, the number of suicides (or circulatory disease-related mortality cases) for each month was considered to be a Poisson count; specifically, the Poisson regression cosinor model for the mean or expected mortality count is as follows,

$$ \log \left[\mathrm{E}\left({\mathrm{M}}_{\mathrm{t}}\right)\right]=\log \left({\mathrm{days}}_{\mathrm{t}}/30\right)+{\mathrm{b}}_0+{\mathrm{b}}_1\sin \left(2\uppi \mathrm{t}/12\right)+{\mathrm{b}}_2\cos \left(2\uppi \mathrm{t}/12\right)+{\mathrm{b}}_3{\mathrm{year}}_{\mathrm{t}}+{\mathrm{b}}_4{\left({\mathrm{year}}_{\mathrm{t}}\right)}^2,\mathrm{t}=1,\dots, 12; $$

where M_t_ is the mortality count in month t. The offset term, log (days_t_/30), is included to adjust the counts for the unequal numbers of days in each month (days_t_). In addition, a quadratic trend for year of observation was included to allow for possible time trends, increases or decreases in the mortality counts over the years. This model efficiently estimates the month of peak mortality incidence, the relative rate (RR) of mortality type during the month of peak incidence compared with the minimum incidence during the whole year, and a 95% confidence interval for the RR. Although the counts of suicide and circulatory disease-related mortality are discernibly different, we note that seasonal patterns of change are modelled on a relative, not absolute scale. Finally, the cross-correlation between monthly mortality counts over time was estimated based on the residuals from the regression of mortality counts on a quadratic trend for year of observation, to adjust for trends in mortality over the years.

## Results

**Fig. 1:**
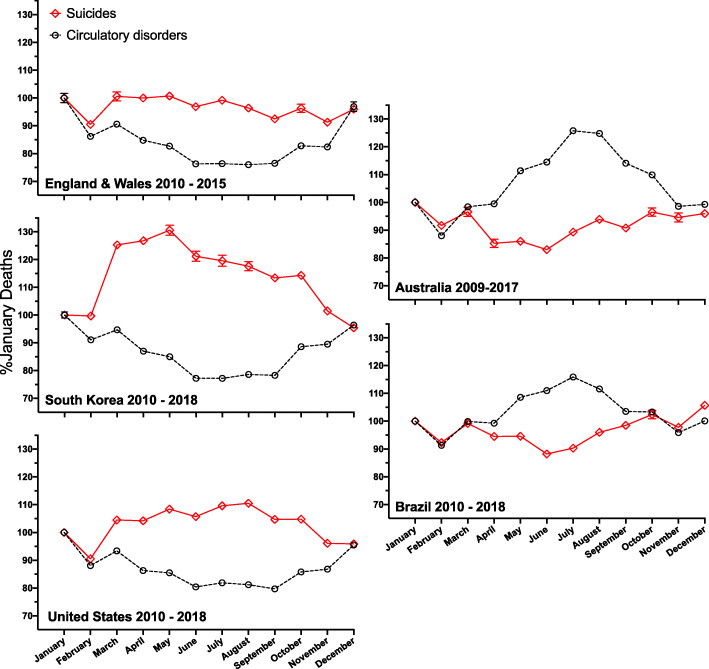
Monthly suicide and circulatory system disease-related deaths in five countries. Legend: Shown are data for three northern hemisphere (left panels: England & Wales, South Korea, United States) and two southern hemisphere (right panels: Australia, Brazil) countries as a percentage of January deaths in each year for each cause. Shown are means (averaged over years sampled) ± standard deviations. These data illustrate that suicide numbers and deaths from circulatory system-related disease increase and decrease, respectively, in spring-summer periods, with inverse patterns for each mortality cause occurring in fall-winter periods. Southern hemisphere temporal patterns are inverted versus northern hemisphere patterns but cross-correlation valences are the same in both hemispheres

Of the five countries sampled, three are in the northern hemisphere (England and Wales, South Korea, and the United States) and two are in the southern hemisphere (Australia and Brazil). Brazil spans the equator but more than 90% of its population lives in the southern hemisphere [23]. Figure 1 illustrates monthly mortality changes in each country across all sampled years, expressed as a percent of total January deaths in all years studied, which adjusts for trends in mortality across years. Temporal trends are similar for men and women (data not shown).

For all five countries, there was evidence of seasonality of circulatory disease-related mortality counts, with relative rate estimates varying from 16 to 29% higher during peak months, and with peak incidence in January in the countries of the northern hemisphere and July in southern hemisphere countries (Table 2). Thus, for circulatory disease-related mortality, we find a remarkably consistent pattern of seasonality, with peak incidence in January in the northern hemisphere and July in the southern hemisphere.

**Table 2:** Estimates of peak and nadir months, rate ratios, and temporal cross-correlation coefficients between mortality types

	Circulatory disease-related			Suicide			
Country	Peak	Nadir	RR (95% CI)	Peak	Nadir	RR (95% CI)	Correl. (95% CI)
Australia	July	Jan.	1.29 (1.26, 1.31)	Dec.	June	1.14 (1.11, 1.18)	−0.32 (−0.44, −0.19)
Brazil	July	Jan.	1.16 (1.14, 1.18)	Dec.	June	1.12 (1.10, 1.15)	−0.57 (− 0.71, − 0.38)
England & Wales	Jan.	July	1.29 (1.24, 1.33)	April	Oct.	1.08 (1.04, 1.12)	0.14 (−0.12, 0.38)
South Korea	Jan.	July	1.29 (1.26, 1.32)	June	Dec.	1.27 (1.21, 1.33)	−0.32 (− 0.42, − 0.20)
United States	Jan.	July	1.22 (1.20, 1.23)	July	Jan.	1.13 (1.11, 1.14)	−0.66 (− 0.75, − 0.56)

Legend: *Jan*. January, *Oct*. October, *Dec*. December; *RR* Rate ratio comparing the relative mortality rate at the peak month to the mortality rate at the nadir month; *Correl*. Cross-correlation between monthly suicide and circulatory system disease-related mortality over time. Note: All analyses have been adjusted to account for the uneven number of days in the month, so that monthly mortality rates have been scaled to an average month of length of 30-days

For all five countries, there also was evidence of seasonality of suicide counts, with relative rate estimates varying from 8 to 27% higher during peak months, and with peak incidence in June or July in two of the three countries of the northern hemisphere and in December in both southern hemisphere countries (Table 2). Interestingly, although there was statistically discernible evidence of seasonality of suicide counts in England and Wales, the relative rate estimate was low, 1.08 (1.04, 1.12), and with peak incidence in the month of April, 2–3 months earlier than in the USA and South Korea.

Finally, in four countries there was evidence of a negative cross-correlation between monthly suicide and circulatory disease mortality over time, with correlations ranging from − 0.32 to − 0.66 (Table 2). That is, there was a negative association between the seasonal counts of suicide and circulatory disease-related deaths. However, there was no statistically discernible evidence of cross-association between suicide and circulatory disease mortality in England and Wales.

## Discussion

We report for the first time using population data from multiple countries, that negative temporal cross-correlations exist between mortality resulting from suicide and from circulatory system-related diseases in four of five countries studied. Temporal cross-correlations ranged from − 0.32 (Australia and South Korea) to − 0.66 (United States). These findings suggest that seasonal factors broadly and inversely moderate temporal patterns for suicide and circulatory system disease-related mortality to different extents in each country. Our null finding for a temporal cross-correlation in England and Wales highlights that the effects we studied are not uniform in all regions; the null effect probably is due in part to a relatively weak seasonal effect on suicide in these areas (Table 2), consistent with prior studies of this region reporting marginal [24, 25] or undetectable [26] effects of season on suicide rates. However, the patterns we detected in 4 of 5 countries in this study are consistent with prior reports of seasonal patterns for circulatory system disease-related mortality [12, 13, 27–29] and for suicide in a number of countries and regions [10, 11, 24, 25, 30–42]. Although we cannot infer from our data whether cross-correlations exist in other countries, studies of temporal patterns of suicide or circulatory disease-related mortality in Canada, Chile, Finland, Greece, Hungary, Iran, Italy, Norway, and Sweden report apparently inverse temporal patterns for suicide [10, 31, 33, 38–45] and for circulatory disease-related mortality [13, 14, 29, 46, 47]. Accordingly, it seems possible that the cross-correlations we identified in 4 countries also exist in other countries.

Seasonally-fluctuating factors that could regulate these temporal patterns are temperature and sunlight. Warm temperatures attenuate risk for circulatory system-related morbidity and mortality [28, 48–57] and warm temperatures, especially abnormally warm temperatures during cool months, increase risk, including short-term risk, for suicide [58–70]. Sunlight similarly moderates circulatory disease-related morbidity and mortality [29, 49, 71–73] and suicide risk [44, 58, 74–79] and may contribute to the temporal cross-correlations we detected. The between-country cross-correlation differences we found thus could result from different patterns of seasonal temperature and sunlight changes in each country.

Since temperature and light exert opposite effects on suicide and circulatory disease-related mortality in several countries, it is plausible that physiologically-adaptive circulatory system responses to heat and light, such as production and release of the bioactive gas nitric oxide (NO), could mediate seasonal effects of heat and light on suicide risk. NO is released as part of thermoregulation, an adaptive process that tightly regulates body heat to prevent heat stress, which can be fatal [80]. Sunlight and artificial light also increase systemic NO levels [72, 81–87] while decreasing blood pressure and vascular resistance [71, 72, 82, 85, 87]. Because high NO and NO-metabolite levels are found in people with histories of suicide attempts [88–90], heat and/or light-induced NO increases could increase risk for suicide. Further, NO-related genes are associated with risk for suicidal behavior [91–94] and with phenotypes relevant to suicide including general psychological distress [95] and depression induced by economic or psychosocial stressors [96]. Additionally, Liu et al. [72] modeled effects of seasonal differences in ultraviolet A (UVA) light, which account for nearly 80% of broadband light-induced NO release from skin, and projected greater light-induced NO release after June-like versus after December-like exposures. Moreover, nitric oxide synthase gene expression fluctuates seasonally [97] as do systemic NO metabolite levels, which are highest in summer and lowest in winter months [98]. Thus, although it is speculative at this point to suggest that NO changes play a role in moderating the effects of season on suicide, evidence from several different sources support this possibility.

Better access to air conditioning and heating systems could blunt some of the seasonal temperature extremes that help drive both mortality types, which could contribute to general declines in these mortality types over time [1, 4]. Psychological stressors including socioeconomic stress, which can increase risk for suicide [99] also increase exhaled NO and blood NO-metabolite levels [100–108]. These findings lend further support to the possibility that high NO levels contribute to suicide risk. It is plausible that socioeconomic and other psychological stressors could combine with seasonal heat and light-induced NO increases to more substantially increase NO levels and suicide risk.

### Limitations

We caution that the estimated peak months, as well as the peak-nadir rate ratio estimates, may be sensitive to the cosinor model assumptions. However, we note that the cosinor model was not adopted for estimation of the temporal cross-correlations. Further, the time-series approach used in this study identified temporal associations but does not provide any information on causal paths. Future studies are required to determine whether the temporal cross-correlations we identified can be found in other regions and can be attributed to NO fluctuations. As noted above, demographic data including socioeconomic, medical, and psychiatric status of decedents are not available in the death registries we sampled and thus these potentially-relevant factors were not considered in this study. Also, our method of analyzing data by month and by country, while revealing clear temporal patterns, likely obscures some temporal effects and regional differences that might otherwise emerge with more granular temporal or geographic analyses (e.g., [38]). Yet, the temporal cross-correlations we revealed are based on hundreds of thousands of cases and while it is possible that the findings may ultimately be found to be artefactual, currently available data from this and other studies suggest that this is unlikely.

## Conclusions

We report negative temporal cross-correlations between mortality resulting from circulatory system-related diseases and from suicide in four of five countries studied, supporting the possibility that seasonal factors that protect against circulatory system-related mortality may increase risk for suicide in some, but not all, countries. The field of suicide prevention urgently needs to identify predictors of suicide risk as current methods are considered to be of little prognostic value [109–111]. Accordingly, although a number of other biological factors likely are involved in this complex disorder [94], we propose that physiologically-adaptive circulatory system responses to heat and light, such as NO production, may increase risk for suicide and should be studied to determine its role in suicide risk.

## Data Availability

Death registry data for Brazil, England and Wales, South Korea, and the United States can be found at the URLs cited in the Reference Section. Death registry data from Australia was obtained in the form of a customized Excel file report that can be provided upon request.

## Abbreviations

CI: Confidence interval
ICD-10: International classification of diseases, tenth revision
NO: Nitric oxide
RR: Relative rate
